# Remote Management of Prosthodontic Emergencies in the Geriatric Population During the Pandemic Outbreak of COVID-19

**DOI:** 10.3389/fmed.2021.648675

**Published:** 2021-07-28

**Authors:** Sami Aldhuwayhi, Saquib Ahmed Shaikh, Amar Ashok Thakare, Mohammed Ziauddeen Mustafa, Sreekanth Kumar Mallineni

**Affiliations:** ^1^Department of Prosthodontics, College of Dentistry, Majmaah University, Al-Majmaah, Saudi Arabia; ^2^Department of Preventive Dental Science, College of Dentistry, Majmaah University, Al-Majmaah, Saudi Arabia; ^3^Department of Pediatric and Preventive Dentistry, Saveetha Dental College and Hospital, Saveetha Institute of Medical and Technical Science, Saveetha University, Chennai, India

**Keywords:** COVID-19, geriatric dentistry, prosthodontics, portable equipment, teledentistry

## Abstract

The global pandemic outbreak of coronavirus disease 2019 (COVID-19) has put the world in a panic situation. It has been evident that the geriatric population is more susceptible to acquire this infection. Thus, due to this ongoing crisis, geriatric individuals cannot visit a dental operatory, and there is the possibility of their dental needs and emergencies to remain unattended. Partial or complete edentulism is very prevalent among the geriatric population, and prosthodontic management of these patients is essential for their well-being. However, the prosthesis can lead to various emergencies of the oral and its supporting structures. There are currently no available recommendations to address these prosthodontic emergencies in geriatric individuals during this pandemic period. Hence, the aims of this minireview were to discuss the common prosthodontic emergencies in the geriatric population and provide recommendations to manage these issues during the COVID-19 pandemic outbreak.

## Introduction

The public health crisis has evolved rapidly with the outbreak of novel coronavirus disease 2019 (COVID-19) in Wuhan, China (1), and exponentially, it has spread to all parts of the world. Healthcare providers are at a higher risk of transmission susceptibility of COVID-19 (2, 3); among them, oral healthcare professionals are at the front line. Oral health problems are more likely to be seen in the geriatric age groups than in the other age groups (4, 5). The geriatric population is prone to increased risk from COVID-19 because of psychological and pathological changes, decreased immune function, and existing comorbidity making them more susceptible to COVID-19 (6, 7). The geriatric population and people with severe underlying medical conditions such as diabetes and cardiac or respiratory diseases are at higher risk of developing symptoms of COVID-19 (1, 6). A Chinese study reported that a case fatality was more in older adults of 50 years of age or above (8). Similarly, an Italian study (9) reported that case fatality was more in geriatric patients (36%) compared with younger patients (15%). On the other hand, Ningthoujam and Khomdram (10) postulated that people with chronic health conditions are at the highest risk from COVID-19 and stated that immunity is the key to defense against COVID-19. According to the Malaysian national oral health survey of adults, it has been reported that 50.8% of the elderly aged 60 years and older geriatric patients had some form of oral prosthesis (11). The majority of prosthodontic procedures require direct contact with the patients and indirect contact with dental technicians and dental laboratories for the procedures which include dental casts, dental impressions, fabrication of removable prosthesis, and mock trials (4, 5). The provision of prosthodontic care in geriatric patients could be urgent and/or emergency, depending on the procedure. Some of the emergencies in prosthodontics include provisional restoration debonding, denture adjustments or repairs, prosthetic fractures, and screw loosening in implant prostheses (12–14). There is an impending need to formulate specific guidelines/protocols for managing the geriatric population in the backdrop of COVID-19, considering the physiological, psychological, and treatment needs of geriatric patients, which are unique (4, 5). Nonetheless, the aims of this minireview were to gain an overview of prosthodontic emergencies in the geriatric population and provide recommendations for their management during this pandemic outbreak of COVID-19.

## Patients' Clinical Handling Protocols

The emergency dental intervention for a COVID-19 suspected patient, when warranted, is that the clinical setup should have negative pressure treatment rooms. The prosthodontist should be aware of the American Dental Association (ADA) recommendations (15). It is mandatory to follow standard airborne and contact protocol by appropriate use of personal protective equipment (PPE) and hand hygiene practices^1^. Prior to the beginning of prosthodontic treatments, 0.23% of povidone-iodine solution mouth rinse for at least 15 s is recommended, and this will help in the reduction of viral load in the patient's saliva (16). The use of single-use and/or disposable instruments and devices is recommended to minimize the cross-contamination of COVID-19 in the dental operatory. The use of a rubberdam will significantly reduce the spread of microbes. Radiographs are generally avoided, and if mandated, the sensor should be double covered to prevent cross-contamination (17). The emphasis on minimally invasive and non-aerosol generating procedures is recommended wherever possible in the dental operatory. The commonly seen prosthodontic emergencies in the geriatric population areas are represented in Table 1.

**Table 1:** Prosthodontic emergency scenarios in the geriatric population and recommendations.

**Type of prosthesis**	**Prosthodontic emergencies**	**Recommendation**
Removable partial denture	If broken or does not fit	• Triaging and teledentistry and suspend the use of the prosthesis to avoid future emergencies
Complete denture	A clean break with reasonably fitting fracture segment	• Triaging and teledentistry and laboratory-based repair by strictly following laboratory biosafety guidelines related to COVID-19
Cantilever bridges resin-bonded fixed partial denture	• Dislodged prostheses usually come out in the hand of a patient • Patient symptomatic with hypersensitivity	• Teledentistry and management after the pandemic period • Application of silver diamine fluoride (SDF) to the exposed vital abutment tooth using the applicator tip
Conventional fixed prosthesis	• Fractured and asymptomatic • Fractured and impinging underlying tissue	• Teledentistry and if asymptomatic the situation can be managed after a pandemic period • Adhering to the American Dental Association, interim guidance for managing emergency dental care and careful removal of the bridge with crown remover
Single or multiple implants supported fixed prosthesis	**Biological emergencies**	
	• Acute peri-implantitis • Asymptomatic failed implants • Symptomatic failed implant	• Pus drainage and debridement under local anesthesia • Managed therapeutically by the AAA protocol (advice, antibiotic, and analgesic) • If symptoms remain, use a local anesthetic to prevent any infection and further bone loss
	**Mechanical emergencies**	
	Screw loosening and screw fracture	• Retightened with the corresponding screwdriver to the correct torque and retrieving the fractured screw portion is laborious and if possible should be postponed till the deferment period
Implant-supported removable prosthesis	**Biological emergencies**	
	Gingival hyperplasia beneath a bar	• Triaging and teledentistry • Temporarily topical astringents in combination with anesthetic can be used for local application and discontinuation of denture until definitive treatment is done
	**Mechanical emergencies**	
	• Loosening of attachments • Fracture of acrylic denture	• Can be retightened with specific screwdrivers at the appropriate torque • A fractured acrylic denture is temporarily repaired with repair resins until a new prosthesis can be fabricated later on

## Emergencies related to prosthesis

### Removable Partial Prosthesis

The partial prosthesis can either be made of acrylic resin, cobalt chrome, or a combination. Dentures can fracture in various ways, including the acrylic part, metal substructure, and/or clasps. The patient might present with emergencies when the broken prosthesis causes noticeable discomfort and impingement to the underlying tissues upon wearing. Prosthodontists are recommended to repair the fracture components by laboratory technicians (previous dental master casts if available or with the help of digital impressions); otherwise, the use of prosthesis is suspended if extensive work is required during this pandemic outbreak of COVID-19 to avoid any further emergency (Table 1).

### Complete Denture Prosthesis

Complete dentures are the most common prosthesis offered to edentulous geriatric patients worldwide (18). Acrylic resin (polymethylmethacrylate) is the material of choice used to manufacture complete dentures, and fracture of the denture is the most common complication associated with it (19). Fracture complete partial dentures may occur due to various reasons such as ill-fit, poor fabrication and design, accidental dropping, parafunction leading to unfavorable stresses, and lack of balanced occlusion (20). Depending on how and where the denture has fractured, the emergency can be managed in various ways. The use of a prosthesis that can cause complications might be avoided till the end of the pandemic period. The virulent effect on acrylic components of the prosthesis is not clearly understood; however, it is better to avoid collecting the prosthesis from the affected geriatric people, and dentists are recommended to send it directly to the dental laboratory for repair. If the denture has a clean break with proper fit and fracture segments are well-located, a laboratory-based repair can be undertaken, provided that the denture is thoroughly disinfected. Sodium hypochlorite exhibited to be the most superior to all the other denture cleansers, and it was suggested that the soaking period is not more than 10 min (21). The dental laboratory technicians should strictly follow the laboratory biosafety protocols (18, 21) and guidelines related to biosafety in surgical pathology (22) while receiving the prosthesis.

### Fixed Partial Denture

Cantilever bridges and resin-bonded fixed partial denture (FPD) that are loosened or dislodged can be managed after the pandemic period if the patient is asymptomatic. The hypersensitivity of exposed abutment tooth can be governed by the self-application of silver diamine fluoride (SDF) or casein phosphopeptide amorphous calcium phosphate (CPP-ACP) to the exposed vital abutment tooth (23). Conventional fixed bridge designs that have dislodged on one abutment or one retainer are challenging to diagnose. The patient is aware of the prosthesis movement, but clinically, it is not easy to diagnose for that reason for dislodgement can be parafunction, poor retention form, resistance form and framework design, and incorrect cementation procedure. If one abutment gets dislodged, it can be assessed by informing the patient and caretaker through video communication to gently tie floss underneath the bridge and pull the prosthesis away from the individual abutment teeth. If the patient is asymptomatic, removing the prosthesis can be made after the pandemic period. If the patient is symptomatic, this condition needs to be attended to, and the patient may consult the dentist either by going to his dental practice or *via* portable dentistry where the dentist can offer his services at the patient's residence. The fractured fixed partial prosthesis can cause emergency conditions if the geriatric patient presents with underlying tissue impingement and pain (24). It leads to disturbance in sleep, anxiety and depression, a decrease in socialization, cognitive dysfunction, multimedications, malnutrition, and increased healthcare costs that affect the quality of life. If the decision to remove the fracture prosthesis is made, the patient should be informed of the findings and discussed options. An appropriate plan should be put in place to ensure the replacement of the fractured prosthesis with temporization. The dental setup should strictly adhere to the ADA interim guidance to manage emergency dental care (24). The precautionary measures that can be taken when dealing with a prosthodontic emergency are as follows:

- Use of anti-retraction functional dental handpiece, four-handed dentistry with high vacuum saliva ejectors.
- Cleaning of handpieces after each patient to remove debris followed by autoclave.
- Prior to treatment, mouth rinse with 0.2% povidone-iodine or 1.5% hydrogen peroxide.
- Frequently clean and disinfect the reception areas, including waiting rooms, door handles, chairs, and washrooms.
- Accompanying persons of the patient should be made to wait outside the reception area or in the car parking.
- Clinic staff should maintain a list of patients who are suspected of COVID-19 infection.
- The following techniques can be used for removing fracture FPD or partially dislodged bridges causing tissue impingement:
- Gentle tapping of the fractured prosthesis using crown removers and avoid using ultrasonic scalers or WAM key to loosen the cement as it generates aerosol.
- Partially dislodged resin-bonded FPD can be removed by placing the sharp chisel's tip.
- The prosthesis should be secured with dental floss held outside the dental assistant's mouth to reduce the risk of inhalation or swallowing the prosthesis.
- FPD can be sectioned with an anti-retraction functional dental handpiece using special diamond cutting burs and removed.

Before planning for removing the fractured prosthesis, the putty index is made to facilitate the fabrication of temporization. Once the dislodged prosthesis is removed, the underlying abutment tooth and tissue are assessed to ensure they are sound and suitable for temporization.

### Single or Multiple Implant-Supported Fixed Prostheses

The often occurring biological emergency after prosthesis fabrication includes peri-implantitis and mobile implant fixtures. Acute peri-implantitis usually presents as painful and fluctuant swelling in the dental implant region, which is an emergency and often is managed by careful debridement under local anesthesia and antibiotic coverage (25, 26). Asymptomatic failed implants can be managed primarily by the AAA protocol (advice, antibiotic, and analgesic) until a substantial diagnosis, and treatment planning should be formulated in the clinical setup after the pandemic period (27, 28). Symptomatic failed implants are recommended to be removed under local anesthesia to prevent further infection and bone loss (29). The common mechanical emergencies include suprastructure prosthesis components such as fixed restorations including lost access cavity restoration and loose implant-supported crown/bridge. In lost access cavity restoration, the patient complains of food entrapment, halitosis, and unesthetic appearance of the prosthesis. Although this situation does not warrant an immediate dental clinic visit, especially during a pandemic outbreak, it can lead to much discomfort to the patient. The patient can procure a temporary restoration on an e-commerce platform such as Amazon, which still allows fast shipment or can be supplied by the dentist. Utilizing teledentistry, the patients' accompanying person can be guided by the dentist. Loose implant-supported crown/bridge mainly happens most commonly because of screw loosening and screw fracture (30). It is classified under emergencies as the patient experiences functional impairment and pain due to the impingement of underlying tissues. Screw loosening can be retightened with the corresponding screwdriver. Worn-out screws are a frequent problem with a screw-retained prosthesis; hence, it is advisable to use a new screw to reduce further fracture risk. The process of retrieving the fractured screw portion is laborious (29) and, if possible, should be postponed. In the event of failure of a luting agent, the previous cement from the crown and abutment can be removed, and the abutment' screw hole can be protected with polytetrafluoroethylene (PTFE) tape or cotton wool, followed by recementation of the crown (30).

### Removable Implant-Supported Prosthesis

Prosthodontic complications commonly occur with a removable implant-supported prosthesis, ranging from loss of retention of attachment systems to loosening of screws (31). Biological emergencies usually include gingival hyperplasia beneath a bar, which can lead to failure of the denture to the seat. It often requires a comprehensive clinical and radiographical examination followed by removal of excessive soft tissue surgically. However, for a temporary measure, topical astringents combined with anesthetic can be used for local application and discontinuation of denture until definitive treatment is done. Mechanical emergencies prevent a patient from using their prosthesis due to loss of retention of the attachment system (25) caused by problems with the components in the denture and fracture of the acrylic denture base. Loose attachments present with significant discomfort for the patients due to the attachment system's mobile male counterpart. It can be simply managed by retightening with specific screwdrivers at appropriate torque. Loss of retention due to worn-out female counterparts of the attachment system usually requires replacing the female component. The replacement procedure is not an emergency and can be postponed until normalcy returns in dental practice. A fractured acrylic denture is another complication that commonly occurs and can be temporarily repaired with repair resins until a new prosthesis can be fabricated later.

### Maxillofacial Prosthesis

Geriatric patients using maxillofacial prosthesis are relatively less compared with other age groups (32). However, the maxillofacial prosthesis significantly impacts the quality of life in the geriatric population (33). The common complications with such prosthesis are loss of retention and prosthesis ill-fit due to surgical site remodeling. Patients who have recently undergone maxillofacial surgery usually have a prefabricated obturator or get a temporary obturator immediately after the surgery in the hospital setup. Such patients can be advised to continue using the same until the pandemic ends (34). A recently inserted definitive obturator may cause discomfort; in such cases, a telephone consultation can help. If the problem persists, it is advised limiting the use of prostheses only while eating. In severe discomfort, it is advised to discontinue the use. Maxillofacial prosthesis requires adhesives for retention, and it can be purchased online. Any other issue related to wear and tear of the prosthesis can be addressed after the pandemic.

### Role of Teledentistry for the Management of Dental Problems in Geriatric Patients

**Figure 1:**
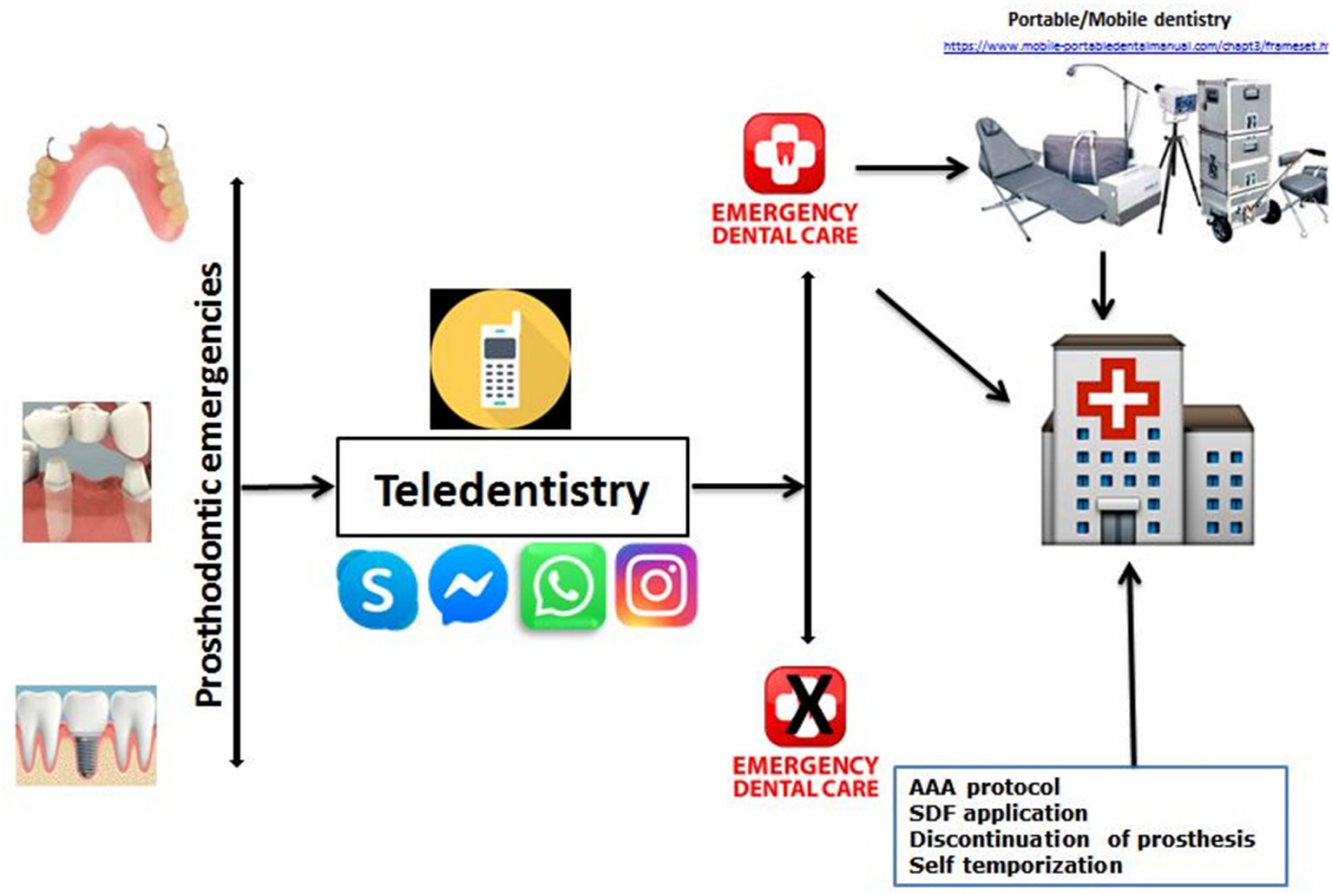
Proposed model for the management of prosthodontic problems in geriatric patients.

It is essential to use a teledentistry model prior to planning an appointment with geriatric patients. A choice can be made to postpone the routine prosthodontic appointment, but the patient needs to be assured and followed up for experiencing discomfort or problems related to their prosthesis. The first step of managing prosthodontic problems encountered in geriatric emergencies during these COVID-19 situations should always be virtual assistance by “WhatsApp” or “Messenger” or “Instagram” or “Skype.” A message with photographs, a video recording, or a video call could be the best option for an initial evaluation in the present COVID-19 pandemic situation (Figure 1). This model was adopted and developed base don using a model proposed recently by Nuvvula and Mallineni (35). The healthcare provider should be trained to use modern web-based communication with patients' assessment using a teledentistry model to avoid potential transmission of COVID-19 (15). After the geriatric patients' virtual screening, it is planned to divide the patients into five groups based on COVID-19 and five groups based on treatment needs. Based on signs and symptoms, patients are divided into five groups (See Footnote 1) [(i) asymptomatic/unsuspected/unconfirmed COVID-19 cases, (ii) symptomatic and suspected/unconfirmed COVID-19 cases, (iii) stable confirmed COVID-19 cases, (iv) unstable confirmed COVID-19 cases, and (v) recovered confirmed COVID-19 cases].

## Discussion

The global pandemic outbreak of COVID-19 has increased the likelihood that the dental care professional should also take precautionary measures to minimize the spread. These measures include careful prescreening of the patient in a confirmed COVID-19 patient is highly obligatory. The common complications are discomfort due to faulty or fractured prosthesis in geriatric patients. On appointments, there may be a higher chance that clinicians might encounter asymptomatic/symptomatic patients. To assess and manage such patients, it might be possible to use a teledentistry model with the suggested questions as described in this short communication. Advancement in technology and social media has made communication easy, and it can be used as an appropriate tool in such pandemic outbreak situations to manage geriatric patients. Furthermore, the use of the recommended questionnaire *via* the teledentistry model has a vital role in approaching geriatric patients (Figure 1). The incubation period of COVID-19 ranges from 2 to 24 days, and most of the patients develop only mild symptoms (15). Consequently, all patients visiting for the management of such emergencies are potential carriers, and the clinicians are advised to follow standard infection control protocols. Radiographs are essential aids in diagnosing dental problems along with clinical examination (36). Radiography should be kept very simple to diminish patient-to-staff contact while providing diagnostic quality radiographs^2^ Intraoral radiographs could be avoided to prevent potential aerosol exposure, and extraoral bitewings were suggested to be appropriate substitutes to the sectional panoramic radiographs as they offer quality images with low radiation (36). However, the authors opine that radiographs are based on essential, but the majority of the emergencies in prosthodontics other than the emergencies related to implants could be solved with recommendations discussed in this minireview. The teledentistry approach plays an essential role in the management of prosthodontic emergencies.

Geriatric patients staying in nursing homes, private homes, or institutions have practical difficulties in reaching the dental office. Henceforth, domiciliary dental care services like mobile dentistry could be used for treating such patients (37–39) by following PPE guidelines (2, 7). In mobile dentistry, the instruments, materials, and equipment can be transferred from the dental office to the patients' location to offer timely care (38). This approach, coupled with meticulous attention to instrument disinfection and application of barrier protection, can be considered a viable treatment option for handling dental emergencies in the geriatric population. Care can be limited to the basic requirements to ensuring that the patient should be pain- and infection-free, which includes treating the emergency, affecting their quality of life (40, 41). Management of biological emergencies, as discussed, can be managed with the help of this portable dentistry approach. Similarly, denture repairs, adding teeth to existing complete or partial dentures, and relining of dentures do not require extensive equipment and can be performed at the patient's residence (40, 41). In this work, the guidelines put forward are general, prosthodontic recommendations, and the final decision will always be the critical insight into the practitioner's judgment to manage geriatric patients during this pandemic outbreak. On the other hand, treatment can be postponed, and pharmacologic management (AAA protocol) for the pain (27, 28) and infection can always be considered (42). The practitioner should always consider the case and treatment categorization based on the recommendations that were suggested. The prosthodontic emergencies in the geriatric population cause an additional burden to those who are already suffering from one or other comorbidities. It is always advisable for the prosthodontist to evaluate these emergencies case by case and use the clinical acumen to aid in decision-making. Therefore, these recommendations help oral healthcare providers treat prosthodontic emergencies in geriatric patients during this pandemic period. Negative ion generators and high-efficiency particulate air (HEPA) filters have also shown a potential reduction of viral load in the dental operatory. The use of these two aids in dental practice may not reduce 100% of the risk of COVID-19 disease transmission in the dental operatory. However, it can assuredly minimize the chance of aerosols generated by dental procedures. In comparison with other dental clinical specialties, very nominal emergency situations occur in the prosthodontics department, and specific treatment could be delayed and does not necessarily need hurried attention. Therefore, the recommendations that are provided in this paper could serve as a guide for older adults with prosthodontic problems and also for general dental practitioners that practice prosthodontics. Consequently, the prosthodontist must visualize the clinic's protective needs as a general dentist and not just as a specialist. This present review gives awareness to the dentists to provide recommendations to prepare them with PPE and the provision of essential emergency treatments to geriatric populations with prosthesis issues.

## Conclusions

The prudent method to manage prosthodontic emergencies in geriatric individuals during a pandemic is to reassure, comfort, motivate, and follow the patient remotely while they are in their home through web-based communications.

Teledentistry and/or mobile dentistry could be considered in the remote management of all prosthodontic non-emergency and emergencies. In emergency cases, teledentistry, along with portable or mobile dentistry, can be recommended with PPE managing geriatric individuals during this pandemic to avoid potential transmission of COVID-19.

## Author Contributions

SA and SS developed the concept. MM, AT, and SM wrote the first draft. SM designed the figure. All authors were involved in reviewing and editing the manuscript.

## Conflict of Interest

The authors declare that the research was conducted in the absence of any commercial or financial relationships that could be construed as a potential conflict of interest.

## Publisher's Note

All claims expressed in this article are solely those of the authors and do not necessarily represent those of their affiliated organizations, or those of the publisher, the editors and the reviewers. Any product that may be evaluated in this article, or claim that may be made by its manufacturer, is not guaranteed or endorsed by the publisher.

## Footnotes

^1^https://www.cdc.gov/coronavirus/2019-ncov/hcp/infection-control-recommendations.html.

^2^https://www.fgdp.org.uk/implications-covid-19-safe-management-general-dental-practice-practical-guide.
